# Habitat Suitability, Distribution Modelling and GAP Analysis of Przewalski’s Gazelle Conservation

**DOI:** 10.3390/ani14010149

**Published:** 2024-01-02

**Authors:** Dongni Liang, Chunwang Li

**Affiliations:** 1 Key Laboratory of Animal Ecology and Conservation Biology, Institute of Zoology, Chinese Academy of Sciences, Beijing 100101, China; liangdongni20@mails.ucas.ac.cn; 2 College of Life Sciences, University of Chinese Academy of Sciences, Beijing 100049, China

**Keywords:** habitat suitability, MaxEnt, GAP analysis, reintroduction, conservation translocation

## Abstract

**Simple Summary:**

The population of Przewalski’s gazelle (*Procapra przewalskii*) has increased over the past decades, but it is still threatened by a variety of environmental factors and human disturbance. Most of the suitable habitats for Przewalski’s gazelle are limited to the vicinity of Qinghai Lake. Moreover, most of the suitable habitat for Przewalski’s gazelle is not included in the scope of the reserve. Thus, conservation translocation may be an effective way of protecting Przewalski’s gazelle.

**Abstract:**

Although the population of Przewalski’s gazelle (*Procapra przewalskii*) has increased, this species is still threatened by a variety of risk factors, such as habitat loss and fragmentation, grassland fencing, grazing conflict, the segmentation of different populations, and declines in population genetic diversity. In order to determine the potential suitable habitat of Przewalski’s gazelle and find a new suitable location for its conservation translocation, we used the MaxEnt model to predict the suitable habitats in Qinghai Province, Gansu Province, and the Ordos Plateau in Inner Mongolia and other regions with historical distribution records. On the basis of the MaxEnt model’s prediction of the potential suitable habitat of Przewalski’s gazelle, we used GAP analysis to determine the existing protection gaps and provide a new reference for the future protection of Przewalski’s gazelle. We found that altitude, temperature, vegetation type, and distance from roads were the main environmental factors affecting the geographical distribution of Przewalski’s gazelle. Most of the suitable habitat of Przewalski’s gazelle is confined around Qinghai Lake. GAP analysis revealed that most of the suitable habitats of Przewalski’s gazelle are not included in the established reserves, and Qinghai Lake National Nature Reserve only covers a small area around Qinghai Lake. The whole reserve only accounts for 7.11% of the area of the suitable habitat for Przewalski’s gazelle and 15.79% of the area of the highly suitable habitat for Przewalski’s gazelle. We suggest that conservation translocation for Przewalski’s gazelle should be put on the agenda. It is necessary to consider reintroducing these gazelles into their potential suitable habitats as a feasible way of establishing new populations and saving this species.

## 1. Introduction

A suitable habitat is crucial for the survival and reproduction of wild animals. At present, some of the most significant threats that wildlife face are the destruction and fragmentation of their habitats, the expansion of human activities and social and economic development, and infrastructure construction, such as roads and railways; these processes have had certain impacts on the activities and habitats of wildlife [1].

Przewalski’s gazelle (*Procapra przewalskii*) is one of the most threatened species. It is an endangered ungulate endemic to the Qinghai–Tibet Plateau and the flagship species in the Qinghai Lake Basin. Przewalski’s gazelle was once widely distributed in China in western Inner Mongolia, Qinghai, Gansu, and Ningxia [2,3]. But due to human population growth, economic growth, the development of animal husbandry, and the large-scale exploitation and utilization of grassland, the ecological environment of the gazelle distribution area has undergone great changes. The population size and distribution area of Przewalski’s gazelle have been shrinking noticeably [4,5].

Recently, the population of Przewalski’s gazelle exhibited a general increase due to protective measures, rising from approximately 200 individuals in 1994 to over 2700 in 2021 [6]. However, this species’ distribution area has not been significantly expanded and remains confined to the vicinity of Qinghai Lake [7]. Despite some progress, threats to Przewalski’s gazelle persist. Global warming is likely to induce changes in the annual average temperature and precipitation, vegetation coverage, vegetation types, and river flow rates in the Qinghai Lake area [8,9]. This may ultimately lead to a decline in habitat quality [8]. In addition, human activities are also increasingly affecting the survival of Przewalski’s gazelle. Examples include increased tourism and overgrazing and the presence of grassland fences that divide the ownership of a grassland [10]. As a result, their habitat is continuously being compressed and fragmented [10,11].

It is therefore crucial to plan for the protection area of Przewalski’s gazelle and consider establishing new populations by reintroducing them to their historical distribution range. Prior to this, an assessment of the species’ suitable habitat is necessary, and Species Distribution Models (SDMs), particularly the Maximum Entropy Model (MaxEnt), are essential tools for studying species distribution and suitability. The MaxEnt model can calculate the distribution probability and possible distribution of species in a predicted area when the entropy is maximum [12,13]. Since the release of the MaxEnt 3.4.4 software product [14], due to its good performance and many advantages, its application in predicting suitable habitats for numerous species has steadily increased [15,16,17]. In addition, conducting further research on conservation gaps for Przewalski’s gazelle is also crucial for this species’ protection. GAP analysis, a geographical approach to conserving biological diversity, involves identifying factors such as vegetation types and species that are underrepresented or absent within a protected area system, with the aim of defining and addressing these gaps [18]. This approach has been widely applied in conservation projects across numerous countries and regions [19,20,21,22].

Hu and Jiang (2011) [9] analyzed the nationwide habitat suitability of Przewalski’s gazelle. In addition to their study, by conducting field surveys and using the latest data, we focus on the prediction and analysis of the historical distribution range and conservation translocation of Przewalski’s gazelle. We combined MaxEnt and GAP analysis for the first time to investigate and predict the suitable habitat for Przewalski’s gazelle in its historical distribution areas, namely, Qinghai, Gansu, Ningxia, and west Inner Mongolia, to determine the suitable habitat and protection vacancy for the survival and reproduction of Przewalski’s gazelle and provide a further basis and reference for the protection planning, management, and conservation translocation of Przewalski’s gazelle.

## 2. Materials and Methods

### 2.1. Study Area

According to research records, Nikolay M. Przhevalsky was the first to collect a specimen of Przewalski’s gazelle in the Ordos Plateau of China in 1875, and Przewalski’s gazelle was once distributed in the Inner Mongolia, Ningxia, Gansu, and Qinghai regions of China [2,23]. From 1995 to 1997, several field investigations were carried out in the Ordos Plateau and its surrounding areas, as well as other areas corresponding to the historical distribution of Przewalski’s gazelle. However, no evidence of living gazelles in Inner Mongolia, China, was found [24]. Therefore, Qinghai, Ningxia, Gansu, and the disputed Ordos Plateau area of Inner Mongolia were included in our research. The terrain and landforms in the study area include mountains, deserts, lakes, and farmlands.

**Figure 1:**
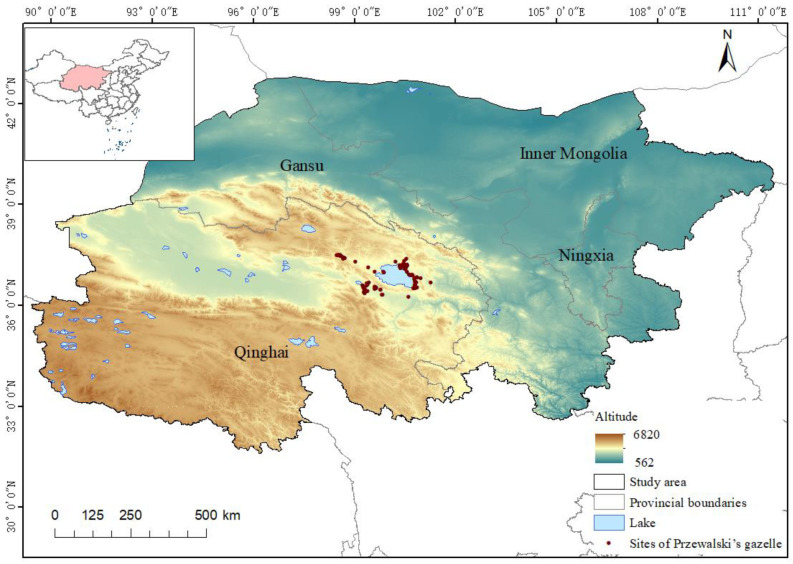
The current distribution sites of Przewalski’s gazelles.

Regarding the current distribution range of Przewalski’s gazelle around Qinghai Lake (36°90′–37°56′ N, 97°50′–101°60′ E) (Figure 1), the average annual temperature is 0.3 °C−1.1 °C, and the annual precipitation is 350–450 mm [7]. The vegetation types include alpine shrub-steppe, alpine meadow, and desert shrub-grassland [7].

### 2.2. Data Collection

We compiled Przewalski’s gazelle presence records from a field survey conducted in 2019 by our research group, data from publications [7], and an online database, the GBIF (the Global Biodiversity Information Facility, http://www.gbif.org, accessed on 21 March 2022) (Table 1). In total, we collected 194 sites of Przewalski’s gazelle. After conducting filtering and screening using ENM Tools (v1.4), duplicate or similar sites were deleted. Finally, 136 sites (all from a field survey conducted in 2019 by our research group) were retained and used for modeling in CSV format according to the requirements of MaxEnt.

The Geographic variables originated from the Geospatial Data Cloud Platform of Computer Network Information Center of Chinese Academy of Sciences (http://www.gscloud.cn, accessed on 3 April 2022). From this website, we obtained data on altitude, rivers, and other information regarding the study area, and we calculated the distance from the roads and rivers using ArcGIS 10.8.

We downloaded 19 climate data (Bio1-Bio19) from the Worldclim database (https://www.worldclim.org/data/worldclim21.html, accessed on 5 April 2022) for the contemporary time period (1970–2000), with a spatial resolution of 30″ and about 1 km [25].

From the Resource and Environmental Science Data Center of Institute of Geographical Sciences and Resources, Chinese Academy of Sciences (https://www.resdc.cn/Default.aspx, accessed on 15 April 2022), we obtained the spatial distribution data of China’s monthly 1 km Normalized Difference Vegetation Index (NDVI) in 2020, which represents the vegetation coverage [26], and downloaded the spatial distribution data of China’s 1:1,000,000 vegetation types on this website, which include grasslands, meadows, deserts, swamps, tundra, evergreen shrubs, deciduous shrubs, evergreen coniferous forests, deciduous coniferous forests, and mixed coniferous forests.

We also downloaded the road layer from the Geospatial Data Cloud Platform of Computer Network Information Center of Chinese Academy of Sciences (http://www.gscloud.cn, accessed on 19 April 2022) and used the Euclidean distance calculation in the toolbox of ArcGIS10.8 to measure distance from roads. From the Wildlife Conservation (WCS) and Center for International Earth Science Information Network (CIESIN) (https://sedac.ciesin.columbia.edu/, accessed on 22 April 2022) websites, we obtained the human footprint index. The population density data for 2020 were downloaded from LanScan website (https://landscan.ornl.gov/, accessed on 23 April 2022).

### 2.3. Environment Variable Filtering

In this survey, there were 28 environmental factors used in MaxEnt v3.4.4, including 19 climatic factors (Bio1–Bio19), 4 geographical factors (Slope, Aspect, Altitude, Dis_river), 2 vegetation factors (NDVI, Veg) and 3 anthropogenic factors (Footprint, Dis_road, Pop) (Table 2).

**Figure 2:**
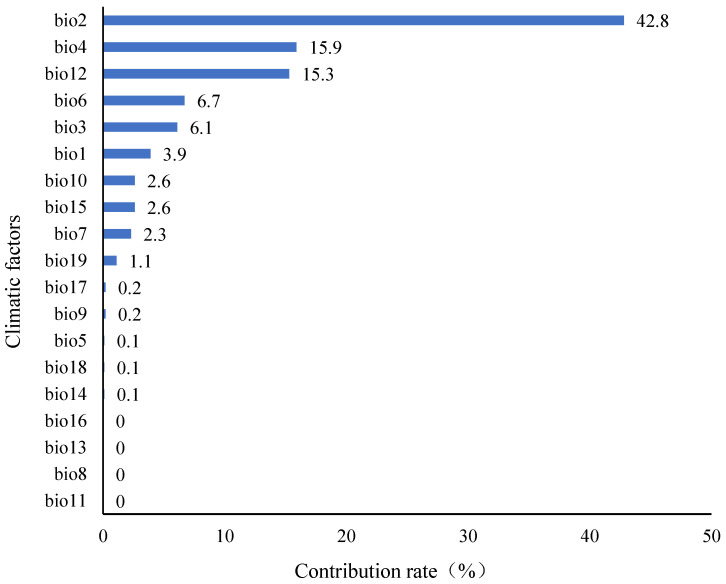
Ranking of contribution rates of climate variables.

In order to reduce the effects of interference and over fitting of multicollinearity among multiple factors on model analysis, all climate variables were pretested, and the contribution rates of all climate variables were analyzed using MaxEnt (Figure 2). In the preliminary analysis, to retain as many variables as possible, we chose 0.1% as the criterion for excluding the contribution rate [27]. That is, after comparing and ranking the contribution rates of various climate variables, the climate variables with contribution rates of less than 0.1% were eliminated. Then, we used ENM Tools software (v1.4) to test the correlation between environmental factors. If the absolute value of the correlation coefficient (r) between the two factors (|r|) was >0.8 [16,28], we removed one of the strongly correlated variables (Table 3). Finally, combined with the ranking of contribution rates and the results of correlation analysis, 7 climate variables were finally selected for the operation of the model, namely, Daily temperature range (Bio2), Seasonal change rate of temperature (Bio4), Lowest temperature in the coldest month (Bio6), Average temperature in the hottest season (Bio10), Precipitation in the driest month (Bio14), Seasonal change of precipitation (Bio15), and Precipitation in the hottest season (Bio18).

As the environmental variable data were grid data that have different resolutions, we resampled variables in arcgis10.8 to 1 km resolution. The projection coordinates were set to WGS1984 UTM Zone 47 N and then converted to the ASCII format required by MaxEnt.

### 2.4. Model Parameter Optimization

Multiple studies have highlighted the importance of considering parameter optimization when utilizing MaxEnt for model analysis, as default parameters may not yield optimal results for different datasets [29,30]. The RM (regularization multiplier) and FC (feature combination multiplier) parameters in MaxEnt can be adjusted to optimize model analysis. There are five selectable features: Linear (L), Quadratic (Q), Hinge (H), Product (P), and Threshold (T). The default parameters in MaxEnt are RM = 1 and FC = LQHP. To evaluate model complexity, using ENMeval, a package in R, we calculated the values of AICc (Akaike Information Criterion with correction) as a measure [31]. Smaller AICc values indicate lower model complexity and greater excellence [32]. In this study, we utilized the “block” method in ENMeval and set RM to 0.5–4 (increasing by 0.5 each time), paired with five feature combinations (FC), namely, L, LQ, LQH, LQHP, and LQHPT. Finally, when the optimal model parameters were RM = 3 and FC = LQHPT, the AICC value was the lowest.

### 2.5. Model Operation

The distribution point data for Przewalski’s gazelle and the 16 environmental variables screened were imported into MaxEnt, and the jackknife operation was used to test the contribution rates of various environmental factors [33]. The response curve function was also selected to determine the relationship between distribution probability and environmental factors. We randomly selected 25% of the sample distribution points as the model test data and 75% as the model training data and set the model so that it would repeat 10 times, with “Cloglog” as the output method.

### 2.6. Result Threshold Division

The distribution prediction data output by MaxEnt were imported into ArcGIS, converted to raster format, and reclassified. To convert data from the continuous suitability index maps to binary habitat and no-habitat maps, a probability threshold is needed to determine potential changes in habitat for species. There are six types of threshold results for the MaxEnt model: (1) minimum training presence threshold; (2) 10-percent training presence threshold; (3) equal training sensitivity and specificity threshold; (4) maximum training sensitivity plus specificity threshold; (5) balance training omission predicted area and threshold value threshold; and (6) equal entropy of threshold and original distributions threshold.

Some studies have proved that “Maximum training sensitivity plus specificity threshold” is the optimal threshold division standard with high accuracy [34]. Therefore, we used this threshold as the division threshold for suitable and non-suitable areas. In this study, our model analysis revealed that this threshold was 0.1985. Then, we used the reclassification tool in ArcGIS10.8 to divide the suitable areas of Przewalski’s gazelle into three grades, namely, non-suitable areas (0–0.1985), suitable areas (0.1985–0.6), and highly suitable areas (0.6–0.99) [35], and calculate and analyze the ecological suitable area changes for Przewalski’s gazelle.

### 2.7. Model Accuracy Evaluation

The accuracy and effectiveness of the prediction results yielded by the model were evaluated using the area under the curve (AUC) value under the receiver operating characteristic curve (ROC). The abscissa of the ROC test curve is 1-specificity, and the ordinate is 1-omission rate. The AUC value is the area enclosed by the ROC curve and abscissa, and its size can represent the accuracy of model prediction results. The larger the AUC value, the more the distribution of species deviates from the random distribution, and the better the prediction effect of the model. The evaluation result of AUC value is not affected by the threshold value, so the evaluation result is more reliable. If the AUC value is above 0.9, this means the accuracy of the model is high and the model is performing well.

### 2.8. GAP Analysis

Based on the obtained habitat suitability distribution map and map of Przewalski’s gazelle reserve, the potential suitable habitat outside the reserve was determined to be the area of protection vacancy.

The data on national and provincial nature reserves were obtained from the Resource and Environmental Science Data Center of the Institute of Geographical Sciences and Resources, Chinese Academy of Sciences (https://www.resdc.cn/Default.aspx, accessed on 6 May 2022).

The data sets consisting of species distribution point, species distribution prediction results, and nature reserve layer used in the GAP analysis process were input into ArcGIS10.8. We utilized the overlay analysis function of ArcGIS to overlay the distribution map of Przewalski’s gazelle obtained from the MaxEnt analysis with the existing protected area layer, aiming to identify the gaps in the protection of Przewalski’s gazelle. These gaps refer to areas that are either unprotected or have weaker protection measures. The potential suitable habitat distribution map generated by MaxEnt was then subjected to Gap analysis with the Qinghai Lake protected area layer. The potential suitable habitats located outside the protected area boundary were considered the protection gap areas.

## 3. Results

### 3.1. MaxEnt Result Accuracy Analysis

**Figure 3:**
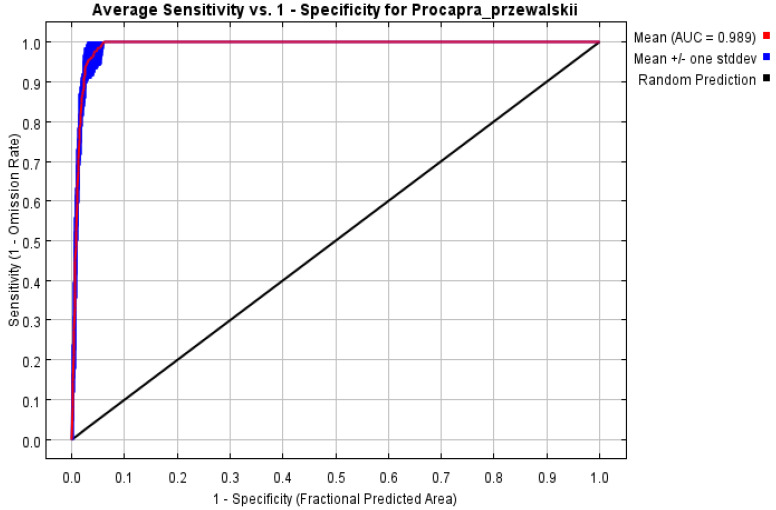
ROC curve of prediction results.

The ROC analysis results showed that the average AUC value of the training set was 0.989, indicating that the potential ecologically suitable area of Przewalski’s gazelle predicted by this model had high reliability (Figure 3).

### 3.2. Main Environmental Variables Affecting the Distribution of Przewalski’s Gazelle

**Figure 4:**
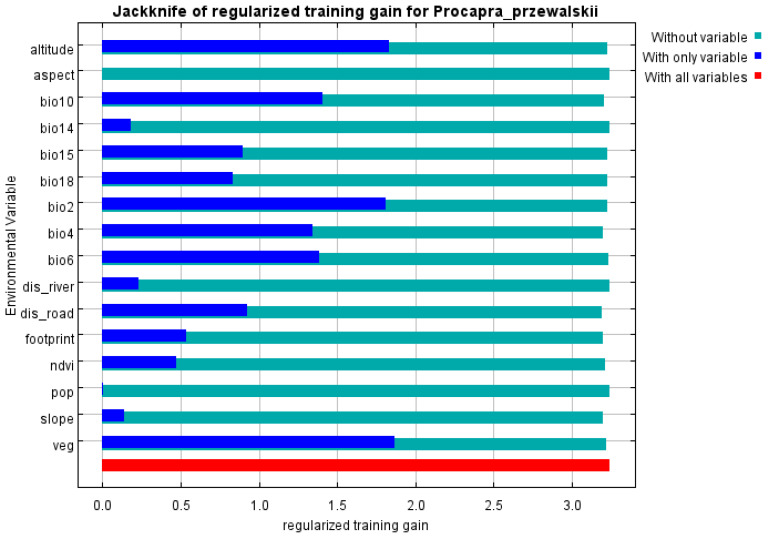
Importance of various environment variables determined using the knife-cutting method.

**Figure 5:**
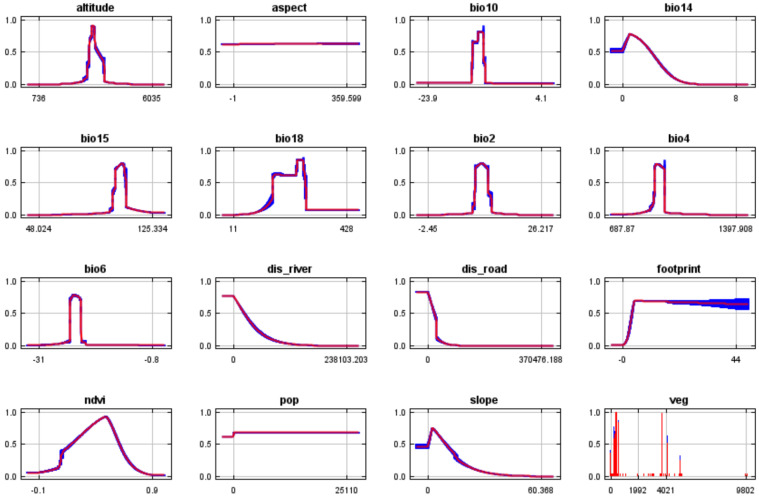
Response curves of environmental variables.

The importance of various environmental variables for the geographical distribution of Przewalski’s gazelle was assessed using the knife-cutting method (Figure 4). The results showed that altitude, vegetation type, daily temperature range (Bio2), seasonal change rate for temperature (Bio4), lowest temperature in the coldest month (Bio6), and average temperature in the hottest season (Bio10) have a great impact on the geographical distribution of Przewalski’s gazelle. It was shown that altitude, temperature, vegetation type, and road distance are the main environmental factors affecting the geographical distribution of Przewalski’s gazelle. According to the response curve of the environmental variables (Figure 5), it can be gleaned that the range of suitable altitude for the survival of Przewalski’s gazelle is about 3000~3400 m. Additionally, the Mean Daily Range (Bio2) for their habitat is between 9 and 12 °C, with the Min Temperature of the Coldest Month (Bio6) ranging between −23 and −20 °C. In terms of slope, the ideal range for this gazelle’s habitat is 2~5°.

### 3.3. Potential Suitable Distribution Area of Przewalski’s Gazelle

**Figure 6:**
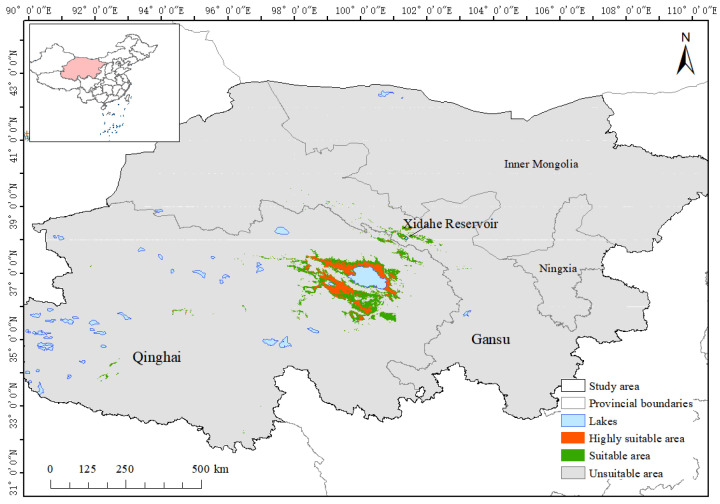
Distribution of potential suitable habitats of Przewalski’s gazelle.

**Figure 7:**
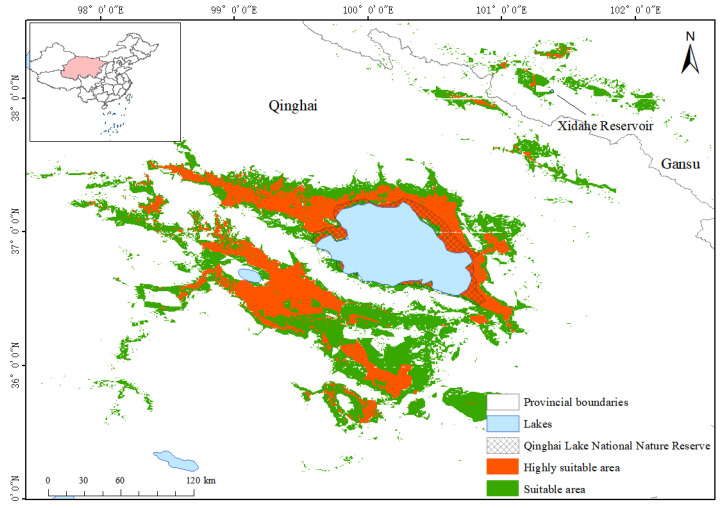
GAP analysis of potential suitable habitat of Przewalski’s gazelle and Qinghai Lake National Nature Reserve.

After we imported the distribution prediction data into ArcGIS10.8 and converted them into raster format, we divided the suitable areas of Przewalski’s gazelle into three levels: non-suitable areas, suitable areas, and highly suitable areas. By calculating the grid area, the highly suitable area of Przewalski’s gazelle was determined to be 11,441.45 km^2^, accounting for 0.61% of the total area of the study area. Furthermore, the suitable area and non-suitable area were 21,506.85 km^2^ and 1,839,440.22 km^2^, respectively, accounting for 1.15% and 98.24% of the total area of the study area. This finding indicates that the highly suitable area of Przewalski’s gazelle is narrow, and most of this area is distributed around Qinghai Lake; another suitable habitat is at the Xidahe Reservoir and its surrounding area (Figure 6 and Figure 7).

### 3.4. GAP Analysis Results

According to the overlay analysis of the layers of highly suitable area, suitable area and Qinghai Lake National Nature Reserve in ArcGIS10.8, the scope of Qinghai Lake National Nature Reserve is small at present, which is only distributed around Qinghai Lake, and most of the suitable habitats of Przewalski’s gazelle are not included in the field of the reserve. We extracted the overlapped part by using the mask and calculated that the protected area accounts for 1529.04 km^2^ of the suitable area, which is 7.11% of the suitable area; The reserve occupies 1806.59 km^2^ of the highly suitable area, accounting for 15.79% of the highly suitable area (Figure 7).

## 4. Discussion

Via MaxEnt modeling, this study identifies altitude, temperature, vegetation type, and distance from roads as the primary environmental factors influencing the geographic distribution of Przewalski’s gazelle. First, altitude and temperature are basic factors affecting species distribution, and this finding is similar to other research results [36]. Additionally, vegetation type is one of the important factors affecting the distribution of Przewalski’s gazelle. Previous researchers have studied the reasons behind the failure of releasing Mohor gazelle (*Gazella dama mhorr*) and found that the suitable habitat for its survival consists of areas with low grass levels rather than areas with high grass levels and a dense canopy [37]. This finding underscores the critical role of vegetation type in the habitat selection of gazelles, presenting significant implications for future relocation efforts. Moreover, distance from roads was identified as another major factor influencing the distribution of Przewalski’s gazelle. Studies have consistently shown that roads can have detrimental effects on species distribution [38]. In the case of Przewalski’s gazelle, roads can impede population connectivity, resulting in habitat fragmentation, decreased quality, and increased mortality for this species [11,39]. Different roads, such as railways, expressways, provincial roads, and township roads, will also have different impacts [40]. As there are Qinghai–Tibet railway and expressways, as well as provincial roads and township roads, in the areas where Przewalski’s gazelle lives, we suggest that further research on how these different roads will affect their distribution is required.

Aside from temperature, climate, and vegetation, slope and aspect were also key environmental factors that concerned and were mentioned by other researchers [27,41], but we found that Przewalski’s gazelle did not seem to have a strong dependence on slope and aspect, which may be due to the small changes in grassland slope and the small impact of slope on grassland vegetation. Meanwhile, some studies have found that different species have different preferences for different slopes in different states (resting or moving) [42]. Besides that, the distribution range of some species also varies with the seasons [43]. This study focused exclusively on the distribution of the gazelle without considering seasonal variations. It is possible that the preferred slope and direction for Przewalski’s gazelle may differ with the changing seasons.

Furthermore, our GAP analysis demonstrated that the Qinghai Lake National Nature Reserve only encompasses a limited range around Qinghai Lake, leaving most of the suitable habitats for Przewalski’s gazelle unprotected. This highlights the limited coverage area of the reserve and suggests the existence of significant protection gaps. In other words, there is still a substantial area of protection gaps with respect to the protection of Przewalski’s gazelle. Despite the rapid increase in the number of nature reserves, certain endangered species still lack effective protection within these designated areas [44]. GAP analysis allows us to identify conservation gaps in a timely manner, making it a valuable tool in conservation ecology. In this study, GAP analysis was conducted based on the suitable habitat of Przewalski’s gazelle. However, further improvements are needed, such as analyzing the local species diversity. To further strengthen the in situ protection of Przewalski’s gazelle in the surrounding areas of Qinghai Lake, it is necessary to scientifically plan the coverage and layout of the Qinghai Lake National Nature Reserve; reasonably divide the protection core area, buffer area, and experimental area; balance the relationship between the tourism industry and the ecological protection of Qinghai Lake; and achieve both the protection of the local ecological environment and biodiversity. At the same time, this will also provide a mutual benefit (win–win results) for the local economy and tourism industry.

In recent years, translocation has been considered an effective conservation strategy for protecting endangered animals [45,46]. Given the ongoing efforts in conservation, conservation translocation is a promising approach to preserving Przewalski’s gazelle. According to the results yielded by the MaxEnt model concerning the distribution area of the Przewalski’s gazelle, we found that the majority of highly suitable habitats were concentrated around Qinghai Lake. It was found that there are minimal suitable habitats for this species in the Ordos region of west Inner Mongolia, China, although researchers have collected specimens of Przewalski’s gazelle in this area [47,48]. Besides the surrounding areas of Qinghai Lake, only a few areas, such as the Xidahe Reservoir and its surrounding area in Gansu, were found to be suitable habitats for Przewalski’s gazelle under the current climatic conditions. This makes Xidahe Reservoir and its surrounding areas crucial for future protection and migration efforts centered on Przewalski’s gazelle. If this site is selected for conservation translocation, it is vital to conduct follow-up investigations (e.g., examining carrying capacity and residents’ attitudes toward Przewalski’s gazelle) in these areas. In addition, this involves conducting cross-provincial assessments and discussions between multiple government departments and experts. For relocation protection, manual means may be needed for relocation, and multiple professional assessments are also required in this regard.

Due to limited time, resources, and capabilities, there are many shortcomings of this study. The limitations of this study include its lack of consideration of livestock density and grazing frequency. Additionally, this study is based on model analysis, which has its own limitations. Further on-site investigations and comprehensive scientific evaluations are needed to address these limitations.

## 5. Conclusions

Our data showed that most of the suitable habitat for Przewalski’s gazelle is not within the scope of the established reserve. Translocation should be considered a feasible way of establishing new populations and saving Przewalski’s gazelle. It is necessary to consider reintroducing these gazelles into an area from which they have disappeared to establish several new populations. Furthermore, although there are still many difficulties in the development of Qinghai Lake National Park [49], the conservation translocation of Przewalski’s gazelle should be considered in China’s national park system.

## Figures and Tables

**Table 1:** Sources of Przewalski’s gazelle presence records.

The Number of Records of Przewalski’s Gazelle’s Presence	Source	Years
13	GBIF	2016–2021
1	[7]	2018
180	This field survey	2019

**Table 2:** Summary of environmental variables.

Types	Variables	Description	Units
Climatic factors	Bio1	Annual Mean Temperature	°C
	Bio2	Mean Diurnal Range (Mean of monthly (max temp-min temp))	°C
	Bio3	Isothermality (Bio2/Bio7) (×100)	%
	Bio4	Temperature Seasonality (standard deviation × 100)	%
	Bio5	Max Temperature of Warmest Month	°C
	Bio6	Min Temperature of Coldest Month	°C
	Bio7	Temperature Annual Range (BIO5-BIO6)	°C
	Bio8	Mean Temperature of Wettest Quarter	°C
	Bio9	Mean Temperature of Driest Quarter	°C
	Bio10	Mean Temperature of Warmest Quarter	°C
	Bio11	Mean Temperature of Coldest Quarter	°C
	Bio12	Annual Precipitation	mm
	Bio13	Precipitation in Wettest Month	mm
	Bio14	Precipitation in Driest Month	mm
	Bio15	Precipitation Seasonality (Coefficient of Variation)	%
	Bio16	Precipitation in Wettest Quarter	mm
	Bio17	Precipitation in Driest Quarter	mm
	Bio18	Precipitation in Warmest Quarter	mm
	Bio19	Precipitation in Coldest Quarter	mm
Vegetation factor	NDVI	Normalized Difference Vegetation Index	-
	Veg	Vegetation Type	
Geographical factors	Altitude	Altitude	m
	Dis_river	Distance from river	m
	Slope	Slope Degree	°
	Aspect	Slope Aspect	-
Anthropogenic factor	Dis_road	Distance from Road	m
	Footprint	Human Footprint Index	-
	Pop	Population density	people/km^2^

**Table 3:** Correlation coefficients (r) of climatic factors.

	bio19	bio1	bio2	bio3	bio4	bio5	bio6	bio7	bio8	bio9	bio10	bio11	bio12	bio13	bio14	bio15	bio16	bio17	bio18
bio19	0	0.59	0.71	0.12	0.45	0.29	0.69	0.57	0.26	0.66	0.40	0.66	0.84	0.71	0.97	0.60	0.73	0.98	0.63
bio1	0	0.00	0.59	0.15	0.30	0.81	0.91	0.42	0.84	0.88	0.89	0.91	0.64	0.57	0.62	0.53	0.58	0.62	0.55
bio2	0	0	0	0.29	0.47	0.23	0.74	0.67	0.33	0.57	0.40	0.67	0.85	0.80	0.76	0.46	0.81	0.75	0.78
bio3	0	0	0	0	0.67	0.51	0.10	0.49	0.48	0.21	0.49	0.16	0.05	0.08	0.18	0.09	0.04	0.16	0.02
bio4	0	0	0	0	0	0.30	0.66	0.97	0.22	0.64	0.17	0.66	0.55	0.46	0.43	0.30	0.51	0.44	0.50
bio5	0	0	0	0	0	0	0.50	0.18	0.96	0.50	0.98	0.51	0.24	0.21	0.33	0.36	0.19	0.32	0.16
bio6	0	0	0	0	0	0	0	0.76	0.56	0.95	0.63	0.99	0.77	0.68	0.72	0.56	0.71	0.72	0.67
bio7	0	0	0	0	0	0	0	0	0.09	0.70	0.02	0.75	0.70	0.61	0.57	0.37	0.66	0.57	0.64
bio8	0	0	0	0	0	0	0	0	0	0.52	0.97	0.56	0.32	0.33	0.33	0.29	0.31	0.32	0.30
bio9	0	0	0	0	0	0	0	0	0	0	0.60	0.97	0.66	0.55	0.65	0.58	0.58	0.65	0.53
bio10	0	0	0	0	0	0	0	0	0	0	0	0.63	0.40	0.37	0.44	0.40	0.36	0.44	0.33
bio11	0	0	0	0	0	0	0	0	0	0	0	0	0.74	0.65	0.68	0.54	0.68	0.68	0.64
bio12	0	0	0	0	0	0	0	0	0	0	0	0	0	0.95	0.86	0.43	0.97	0.86	0.94
bio13	0	0	0	0	0	0	0	0	0	0	0	0	0	0	0.73	0.21	0.99	0.73	0.98
bio14	0	0	0	0	0	0	0	0	0	0	0	0	0	0	0	0.60	0.75	0.99	0.66
bio15	0	0	0	0	0	0	0	0	0	0	0	0	0	0	0	0	0.27	0.61	0.20
bio16	0	0	0	0	0	0	0	0	0	0	0	0	0	0	0	0	0	0.75	0.98
bio17	0	0	0	0	0	0	0	0	0	0	0	0	0	0	0	0	0	0	0.66
bio18	0	0	0	0	0	0	0	0	0	0	0	0	0	0	0	0	0	0	0

Note: The different colors show different degrees of correlation. 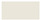 corresponds to r ≤ 0.10, 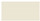 corresponds to 0.10 < r ≤ 0.20, 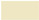 corresponds to 0.20 < r ≤ 0.30, 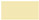 corresponds to 0.30 < r ≤ 0.40, 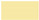 corresponds to 0.40 < r ≤ 0.50, 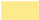 corresponds to 0.50 < r ≤ 0.60, 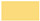 corresponds to 0.60 < r ≤ 0.70, 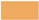 corresponds to 0.70 < r ≤ 0.80, 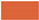 corresponds to 0.80 < r ≤ 0.90, and 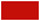 corresponds to r > 0.90.

## Data Availability

The data presented in this study are available on request from the corresponding author. The data are not publicly available due to government policy restrictions.
